# Enzymes for Pancreatic Islet Isolation Impact Chemokine-Production and Polarization of Insulin-Producing β-Cells with Reduced Functional Survival of Immunoisolated Rat Islet-Allografts as a Consequence

**DOI:** 10.1371/journal.pone.0147992

**Published:** 2016-01-29

**Authors:** Paul de Vos, Alexandra M. Smink, Genaro Paredes, Jonathan R. T. Lakey, Jeroen Kuipers, Ben N. G. Giepmans, Bart J. de Haan, Marijke M. Faas

**Affiliations:** 1 Immunoendocrinology, department of Pathology and Medical biology, University of Groningen, Hanzeplein 1, 9700 RB Groningen, The Netherlands; 2 Department of Surgery and Biomedical Engineering, University of California Irvine, Orange, CA, 92868, United States of America; 3 Department of Cell Biology, University Medical Center Groningen, University of Groningen, P. O. Box 196, 9700 AD, Groningen, The Netherlands; INSERM UMRS 1138, FRANCE

## Abstract

The primary aim of this study was to determine whether normal variations in enzyme-activities of collagenases applied for rat-islet isolation impact longevity of encapsulated islet grafts. Also we studied the functional and immunological properties of rat islets isolated with different enzyme preparations to determine whether this impacts these parameters. Rat-islets were isolated from the pancreas with two different collagenases with commonly accepted collagenase, neutral protease, and clostripain activities. Islets had a similar and acceptable glucose-induced insulin-release profile but a profound statistical significant difference in production of the chemokines IP-10 and Gro-α. The islets were studied with nanotomy which is an EM-based technology for unbiased study of ultrastructural features of islets such as cell-cell contacts, endocrine-cell condition, ER stress, mitochondrial conditions, and cell polarization. The islet-batch with higher chemokine-production had a lower amount of polarized insulin-producing β-cells. All islets had more intercellular spaces and less interconnected areas with tight cell-cell junctions when compared to islets in the pancreas. Islet-graft function was studied by implanting encapsulated and free islet grafts in rat recipients. Alginate-based encapsulated grafts isolated with the enzyme-lot inducing higher chemokine production and lower polarization survived for a two-fold shorter period of time. The lower survival-time of the encapsulated grafts was correlated with a higher influx of inflammatory cells at 7 days after implantation. Islets from the same two batches transplanted as free unencapsulated-graft, did not show any difference in survival or function *in vivo*. Lack of insight in factors contributing to the current lab-to-lab variation in longevity of encapsulated islet-grafts is considered to be a threat for clinical application. Our data suggest that seemingly minor variations in activity of enzymes applied for islet-isolation might contribute to longevity-variations of immunoisolated islet-grafts.

## Introduction

The immunoprotection of pancreatic islets has been proposed to be an efficacious technology for transplantation of islets in the absence of application of immunosuppression. In the past decade major advances have been made with this technology [1]. Human trials demonstrated temporary but reproducible functional survival of microencapsulated pancreatic islet grafts in human diabetic patients [2]. Also it has been shown that microencapsulation may contribute to solving the shortage of donor tissue as prolonged survival of xenotransplanted islet grafts has been demonstrated in both chemically induced and autoimmune diabetic rodents [3], dogs [4], and monkeys [5]. Despite all these successes a persistent, fundamental barrier has to be overcome since graft survival varies considerably from lab-to-lab from several days to months [6].

The variations in survival rates are usually interpreted to be the consequence of either inflammatory responses against the capsules or to be associated with limitations of the transplantation site such as the low oxygen tension in the peritoneal cavity [7]. The purity of the alginate [8, 9], the interaction of the alginate with the polyamino acid [10–12], the mechanical stability [13, 14], and also physicochemical changes of the capsules after implantation [15] are considered to be crucial factors in the responses against the capsules. The absence of direct access to the vasculature may also interfere with islet survival [7].

In transplantation of free nonencapsulated islets, the role of the quality of the islets in longevity of the grafts has gained much attention [16, 17]. Surprisingly, this has not been studied for immunoisolated grafts while many try to identify the factor contributing to success or failure of immunoisolated islet-grafts [18–24]. Before transplantation, islets are isolated from the pancreas by enzymatic digestion of the exocrine tissue [25]. The enzyme preparations used for this procedure vary from lot to lot, and as a consequence also the quality and the immunogenicity of the islets may vary [25, 26]. Rat-islet isolation is a process that is highly standardized and based on thousands of isolations performed over a period of more than two decades [26]. Standards have been set for the activities of the applied enzymes [26]. Within these activities rat-islets can be isolated without impacting the functional survival [26]. The enzymes applied for islet isolation are called collagenases but in fact are composed of a mixture of collagenases, neutral proteases, and clostripains [26]. The activities of these enzymes are monitored and documented for two decades in our laboratory [26] and used for transplantation studies of free islet grafts and encapsulated islet grafts. As some unexplained variation in survival was noticed with encapsulated islet grafts, we decided to study and compare the impact of collagenase-batches. The collagenases applied in this study are recommended for islet isolation by the vendor and within accepted boundaries of collagenases, neutral proteases, and clostripains activities for rat islet isolation [26], and do not contain significant amounts of endotoxins. The effects of these collagenases on islet quality, on immunogenicity, and on applicability for encapsulated and free-islet grafts were studied. Islet quality was assessed by a relative new technique named nanotomy. This technique combines large-scale high-resolution EM data allowing examination of ultrastuctural features of islets such as macromolecules, cell-cell contact, polarization of endocrine cells and other organelles in the islets. On purpose we avoided extreme differences in enzyme activities as it was our aim to investigate whether commonly accepted variations in yield and quality contribute to survival of immunoisolated grafts [26]. Only highly purified alginates that do not provoke inflammatory responses were applied.

## Materials and Methods

### Graft recipients and donors

Pathogen-free inbred male Albino Oxford rats weighing 300–320 grams served as islet recipients. Pathogen-free inbred Lewis rats or Albino Oxford rats weighing 300–350 grams served as islet donors. All experimental animals were obtained from Harlan (Horst, The Netherlands). The animals were fed standard rat chow and acidified water *ad libitum*. All animal experiments were performed after receiving approval of the institutional Animal Care Committee of the Groningen University and all animals received human care in compliance with the Dutch Law on Experimental Animal Care.

Diabetes was induced in the AO-recipient rats by injection of 75 mg/kg streptozotocin (Zonasar, Upjohn Co., Kalamazoo, MI) in the tail vein. Only animals showing weight loss and two constitutive blood glucose measurements exceeding 20 mmol/L over a period of two weeks served as islet graft recipients. The glucose concentration was determined using the Accu-Chek Sensor system (Roche, France). After transplantation of free AO-islets or encapsulated Lewis-islets, non-fasting blood glucose levels were determined in blood sampled from the tail vein once every two days. Two blood glucose levels exceeding 20 mmol/L in a two week period was considered as reestablishment of diabetes and complete islet graft failure.

### Collagenases

Crude *C*. *Histolyticum* collagenases are composed of two types of collagenase enzymes and several proteases [27]. We always applied collagenase type XI (Sigma, St Louis, MO). On the basis of more than two-thousand islet isolations with more than fifty different lot numbers of collagenases we have defined activity-limits for furylacryloyl-Leu-Gly-Pro-Ala (FALGPA) hydrolysis, collagen digestion, neutral protease activities, and clostripain in order to allow for adequate isolation of rat islets [26]. Commercial collagenases with other activity were associated with either dramatic low yields or nonfunctional islets. The collagenases applied in the present study were purchased from Sigma. The activities are listed in Table 1. Endotoxin levels were < 0.006 ng/mg as measured by the Limulus amebocyte lysate (LAL) assay.

**Table 1:** Enzymatic activities of commercial collagenases applied for rat-islet isolation. Activities are provided by the manufacturer (Sigma) for the two lot-numbers collagenase type XI.

Enzyme	Activities	
	Lot 1	Lot 2
Collagenase (FALGPA hydrolysis)	3.1	4.1
Collagenase (Collagen digestion)	1370	1730
Neutral protease (Caseinase)	16	44
Clostripain	1	1.65

### Islet isolation

Islets were isolated as described before [26]. The pancreas was distended via the ductus with Krebs-Ringer-Hepes (KRH) buffered with 25 mM Hepes containing 10% (weight/volume, w/v) BSA without collagenases. After distension, the organ was chopped into pieces of 1 mm^2^. The chopped pancreas was incubated at 37°C with 1.0 mg/ml of collagenase with KRH containing 10% BSA. After 10 minutes the tissue fragments was washed and sedimented twice. Tissue fragments were subsequently incubated at 37°C with 0.7 mg/ml of collagenase with KRH containing 10% BSA. After 8 minutes the tissue fragments the digest was allowed to sediment and washed twice with RPMI containing 1% BSA. [26]. For quantification, all islets were collected [28], followed by a measurement of the diameters of islets in a 4% aliquot of the islet suspension. For diameter-measurements we applied a 25x magnification dissection microscope (Bausch and Lomb BVB-125, and 31-33-66) equipped with an ocular micrometer.

### Insulin secretion during glucose challenge

In static incubation experiments, Lewis-islets were tested in four separate samples of 10 islets each. To minimize the variability of the mean insulin responses, we meticulously selected islets with diameters between 150 and 200 μm. The islets were preincubated for 45 minutes in 2 ml Krebs-Ringer-bicarbonate (KRB), gassed with 95% O_2_ and 5% CO_2_, containing 0.25% BSA and 2.75 mM glucose. The quantitative insulin secretion was then assessed by three consecutive incubations of (i) 45 min in 2.75 mM glucose in KRB, (ii) 45 min in 16.5 mM glucose in KRB, and (iii) 45 min in 2.75 mM glucose in KRB. At the end of each incubation period, the incubation media were removed and frozen for insulin determination. The insulin secretory responses were expressed as nanogram of insulin.ml^−1^.10 islets^−1^.45 min^−1^.

### Encapsulation

Due to the laborious nature of the procedures only two transplantations could be performed on a single day. Per day, islets were isolated from rats with application of the two collagenases. Pancreata and islets were treated with the two collagenase lots but the islets were further treated with the same media and reagents. The pancreata and islets were treated exactly the same but were kept separately. After one day of culturing (CMRL1066 supplemented with 10% FBS), the islets were encapsulated in two encapsulation runs but with the application of the same procedure, media, and polymers. The sequence of encapsulation was switched for the two collagenases on constitutive days. On each day one animal received an islet graft under the kidney capsules or as encapsulated graft in the peritoneal cavity. Thus each animal received a graft from a separate isolation procedure. Transplantation was only performed when the aimed endocrine volume of 10 μl was collected.

Alginate was obtained from ISP Alginates Ltd UK and purified as previously described [29]. Alginates always had an endotoxin content which was < 0.006 ng/mg as measured by the Limulus amebocyte lysate (LAL) assay. Alginates were dissolved at 4°C in Krebs-Ringer-Hepes (KRH) with an appropriate osmolarity. Islets were mixed at a concentration of 1000 islets/ml by very gentle agitation. The alginate solution was subsequently converted into droplets using an air-driven droplet generator as previously described [30]. Polylysine-alginate encapsulation was performed as described elsewhere [31, 32]. The capsules had a diameter of 500–650 μm. The same microscope was used for inspection of the capsules prior to implantation.

### Implantation and explantation of islets and capsules

Transplantation of the islet isografts under the kidney capsule was performed immediately after the AO-islet isolation procedure. From each islet isolation batch one grafts was used as free islet grafts under the kidney capsules and the other as encapsulated graft to exclude day-to-day variations. Only male donors of 300–315 gram were applied.

Grafts were composed of 10 μl, i.e. the equivalent of the endocrine volume of the rat pancreas [33] which equals 700 to 1100 islets. Transplantation under the kidney capsule was performed at the upper pole of the kidney.

Encapsulated Lewis-islets were injected into the peritoneal cavity with a 16 G cannula via a small incision (3 mm) in the linea alba. The implanted volume was always 2.0 ml as assessed in a syringe with appropriate measure.

For studying the inflammatory responses, animals were subjected to laparatomy after graft failure. Microcapsules were either free-floating and non-adherent, or adherent to the surface of abdominal organs. First, non-adherent microcapsules were retrieved by peritoneal lavage, and brought into a syringe with appropriate measures for quantification of the retrieval rate [34]. Briefly, peritoneal lavage was performed with 40 ml KRH. The lavage was collected in a 50 ml tubes. After sedimentation the capsules were brought in a 2 ml syringe after which the retrieval volume was determined as previously described [34]. Subsequently, the microcapsules adherent to the surface of abdominal organs, were excised and processed for histology.

All surgical procedures were performed under sterile conditions using fluothane/oxygen anesthesia with vacuum aspiration.

### Intravenous glucose tolerance tests

Three weeks after transplantation, all animals received a permanent silicon catheter in the right vena jugularis [35], for blood sampling and infusion in unanesthetized, undisturbed, and freely moving animals.

Four weeks after transplantation intravenous glucose tolerance tests (IVGTT) were performed. This was always done after a two hour fasting period. IVGTTs were performed by infusion of 200 mg glucose at a rate of 10 mg/min. Blood samples were taken immediately before time point 0, and at 1, 3, 5, 7, 10, 15, 20, 25, 30, 45, 60, 75, 90, 105, and 120 min after the start. Sampled blood was replaced by donor blood. In some recipients, glucose tolerance could not or not completely be tested as a consequence of technical complications.

Glucose concentrations during the glucose tolerance testing were determined in whole blood by a ferricyanide method with a Technicon autoanalyser. Plasma insulin was measured by a radioimmunoassay (Linco, Rat Insulin Ria Kit, St. Charles, MO, USA).

### Luminex

In some experiments freshly isolated islets were cultured for 24h to quantify release of chemotactic cytokines and chemokines. Cytokine levels in the supernatant were measured using a MilliPlex^™^ premixed cytokine assay, according to the manufacturer’s instructions (Linco Research Inc, MO, USA). This customized kit simultaneously measures the following molecules: MCP-1, IP-10, GRO, MIP-1, MIP-2, RANTES, NGF, and VEGF.

### Microscopy

To assess the integrity of capsules before implantation, samples of capsules were meticulously inspected for the presence of irregularities or broken parts in the capsule membranes using a dissection microscope.

To detect physical imperfections and to assess the composition and degree of overgrowth after implantation, samples of adherent capsules recovered by excision and samples of non-adherent capsules were fixed in pre-cooled 2% paraformaldehyde, buffered with 0.05 M phosphate in saline (pH 7.4), and processed for glycol methacrylate (GMA) embedding [36]. Sections were prepared at 2 μm and stained with Romanovsky-Giemsa stain and applied for detecting imperfections in the capsule membrane and for quantifying the composition of the inflammatory overgrowth and determining the number of capsules with and without overgrowth. Different cell-types in the overgrowth were assessed by identifying cells in the capsular overgrowth with the morphological characteristics of monocytes/macrophages, lymphocytes, granulocytes, fibroblasts, basophiles, erythrocytes, and multinucleated giant cells. To confirm the adequacy of this approach, portions of adherent and non-adherent capsules were frozen in precooled iso-propane, sectioned at 5 μm, and processed for immunohistochemical staining and quantification of the different cell types as previously described [37]. The monoclonal antibodies used were: ED1 and ED2 against monocytes and macrophages, HIS-40 against IgM bearing B-lymphocytes, and R73 against CD3^+^ bearing T-lymphocytes. In control sections we used PBS instead of the first stage monoclonal antibody. Quantification of these cells types after immunocytochemistry were compared with the assessments on the basis of morphological markers and always gave similar results.

For large-scale EM studies (nanotomy), islets were fixed in 2% paraformaldehyde (PFA) and 0.5% glutaraldehyde (GA) in 0.1 M cacodylatebuffer for minimal 1 hour and pelleted in 2% low melting point agarose [38]. Agarose embedded islets were post fixed for minimal 2 hours with 1% osmiumtetroxide and 1.5% potassiumferrocyanide in 0.1 M cacodylate buffer at 4°C. After dehydration with a graded series of ethanol and a subsequent incubation of 30 min in acetone, islets were rotated overnight in 1:1 mixture of acetone and EPON epoxy resin, and embedded in pure EPON resin and polymerised at 58°C. Ultrathin sections (60 nm) were cut on an ultramicrotome with a diamond knife (Diatome Inc., Biel, Switzerland) and collected on 75 mesh copper TEM grids. Heavy metal contrasting was done using 2% uranylacetate in methanol followed by Reynolds lead citrate. All nanotomy data were obtained using a Zeiss Supra 55 in STEM mode at 29KV. Mutliple tiles covering large areas, were recorded using ATLAS scan generator and software (Fibics, Canada). Stitching of the tiles was done using VE viewer (fibics, Canada). VE viewer was also used to convert the data into html which can be assessed at www.nanotomy.org/. The data were also converted in to a single TIF file (downscaled to 10 nm pixel size) using VE viewer. Annotation has been performed manually on these TIF files using Adobe Photoshop CS6 based on structural difference between the different islet-cell populations [38].

### Statistical analysis

Normal distribution of the data was confirmed using the Kolmogorov-Smirnov test. Statistical comparisons were performed using the one-way ANOVA. Data were expressed as mean ± standard error of the mean (SEM). *p*-values <0.05 were considered to be statistically significant.

## Results

### Functional, ultrastructural, and immunological differences in collagenase-isolated pancreatic islets

To determine the efficacy of the enzyme lots in isolating islets, we determined the yield, the function, and the ultrastructural-morphology by a new technique that allows unbiased acquisition, examination and datasharing of isolated islets using large scale EM, also know as nanotomy [38]. Also we determined the release of proinflammatory chemokines by the islets.

Lot 1 collagenase was associated with a 30% higher yield (*p* < 0.05) in number of islets. The concomitant, calculated endocrine volume of the isolated islets showed a less dramatic difference in yield (Table 2).

**Table 2:** Number and endocrine volume of islets isolated from an AO/G rat. Values represent mean ± SEM.

Enzyme	n	numbers	endocrine volume (μl)
Lot 1	5	256 ± 21	2.5 ± 0.2
Lot 2	5	174 ± 23	2.2 ± 0.1

The more pronounced difference in number than in volume of the islets should be attributed to differences in size of the islets after isolation. Islet batches isolated with lot 2 contained a trend of more islets with a diameter of 300 μm and higher and statistically significantly less islets with a diameter of 120 μm and smaller (*p* always <0.05) (data not shown).

**Fig 1:**
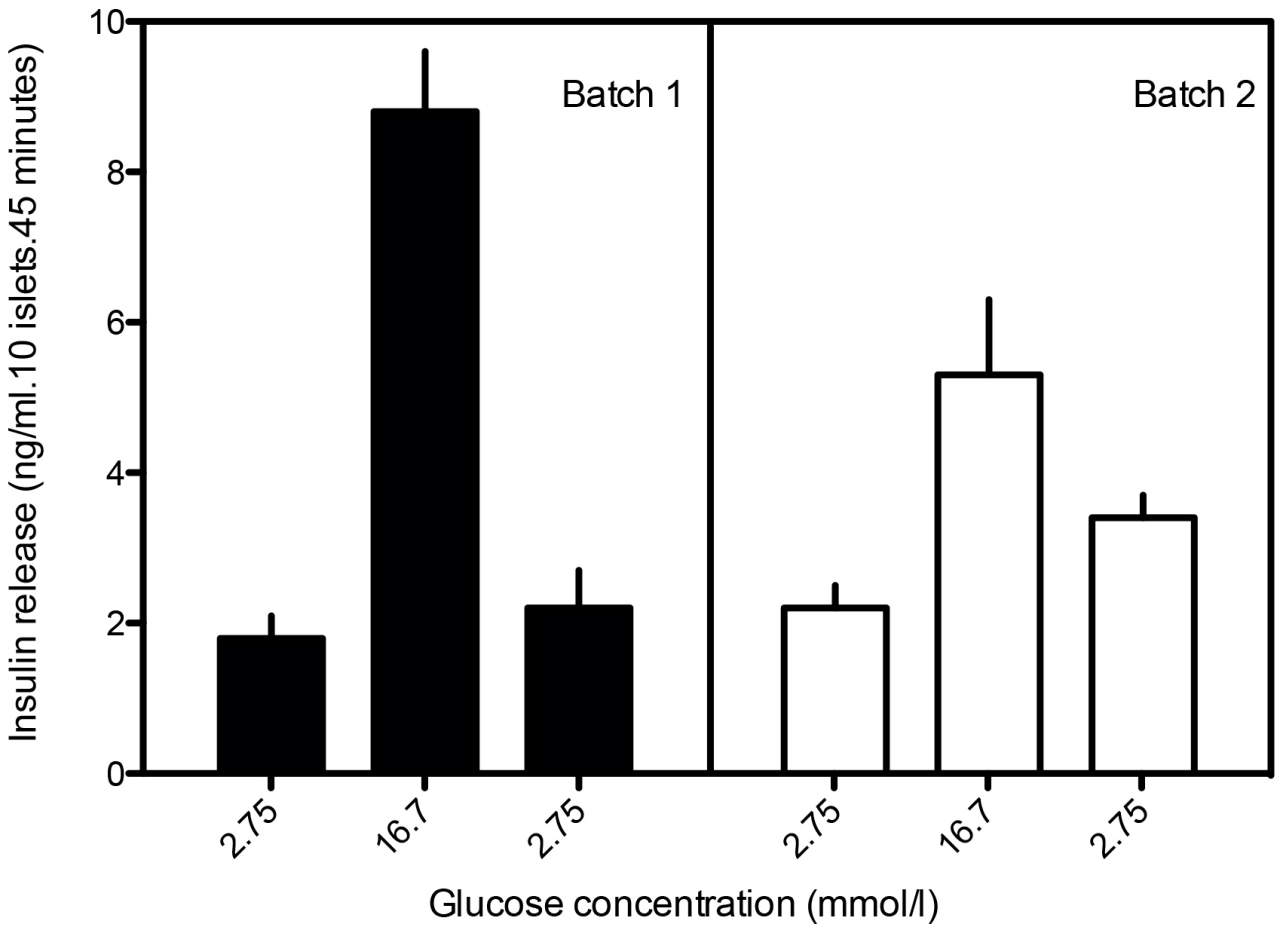
Quantitative insulin response after glucose challenge (after static incubation) of AO/G rat islets after isolation with two different types of collagenases. Enzyme activities are mentioned in Table 1. All experiments were repeated five times. Values represent means ± SEM.

Not only the yield but also the function of the islets was dependent on the enzyme preparation applied. The stimulated insulin secretion (during challenge with 16.7 mM glucose) was higher with batch 1 than with batch 2 (*p* < 0.05) (Fig 1). Also a less adequate return to basal insulin secretion was observed with batch 2 when compared to batch 1 (*p* < 0.05). The results shown are for Lewis-islets but similar results are obtained and available for AO-islets.

**Fig 2:**
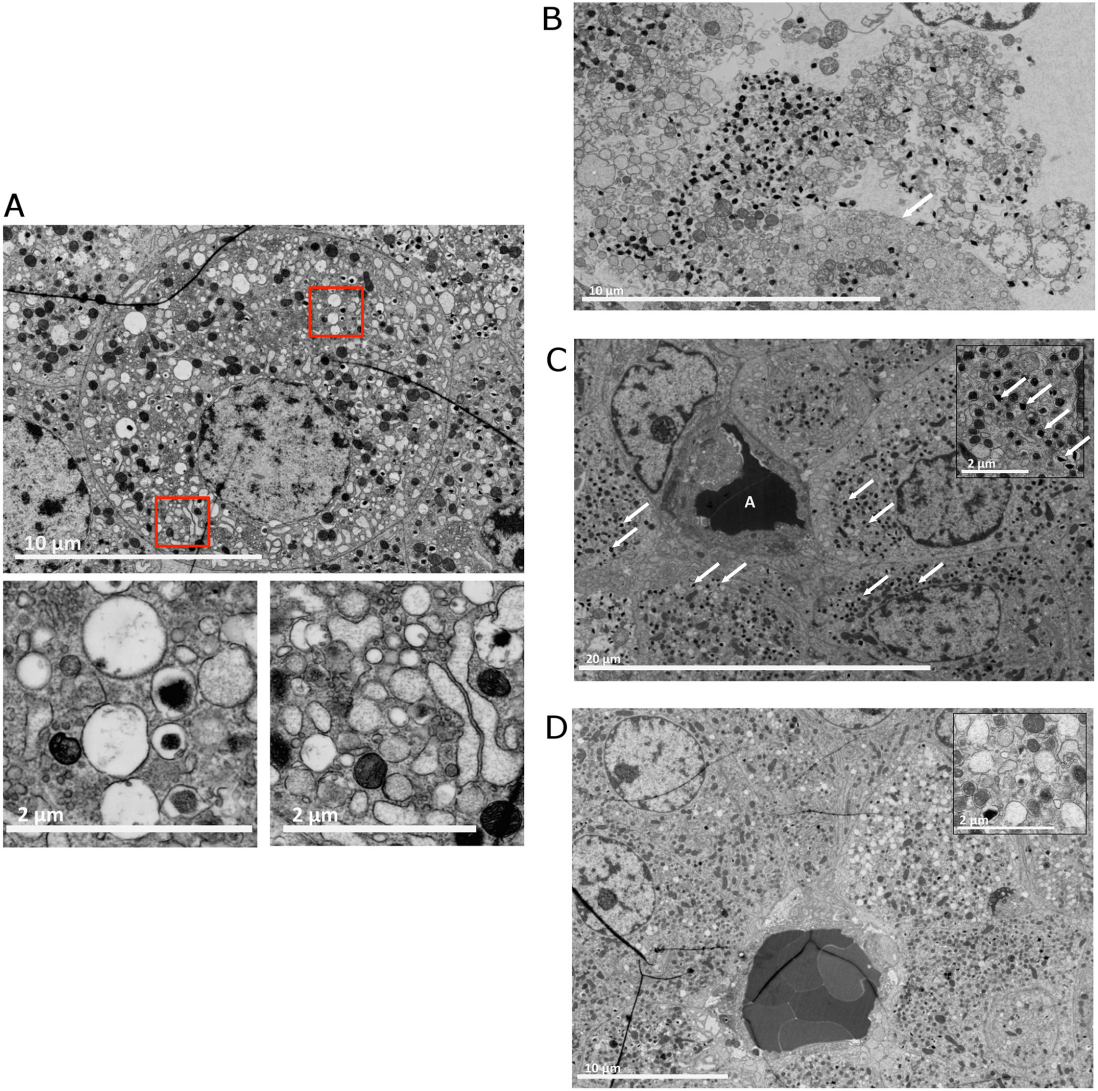
Nanotomy of collagenase-isolated pancreatic rat islets. (A) A cell with severe stress. The red-boxed areas show swollen vesicles and dilated stressed ER. These vesicles can be desintegrated mitochondria or empty insulin granula indicating a lack of insulin production. (B) During the isolation process the enzymes can disintegrated the cell-membrane causing the organelles and insuline granules to float around in remnants of cytosol, not being contained by a cell membrane. The arrow indicates an intact cell membrane. (C) An islet isolated with batch 1 collagenase. The structure with A depicts a capillary. The β-cells surrounding the capillary are well granulated with insulin granula (the arrows indicate a few classical examples, see insert). The β-cells are polarized. The majority of granula can be found on the site of the β-cell adjacent to the blood vessels. For full resolution, visit http://www.nanotomy.org/deVos/2013-319a/1.html. (D) An islet isolated with collagenase batch 2. The β-cells contain much less insulin granules (see insert) than the batch 1 β-cells and are not polarized. Note that the few granula are found scattered throughout the cell. For full resolution, visit http://www.nanotomy.org/deVos/2013-320a/1.html.

To correlate the loss of functionality with possible ultrastructural changes induced by the collagenase we applied recently developed nanotomy [38]. Nanotomy generates large-scale high-resolution EM data allowing examination of macromolecules or organelles in the islets. Islet preparations contained cells with a compromised ultrastructural morphology such as characteristic swollen vesicles (Fig 2A). The most severe signs of stress were cells lacking or only having a partial cell membrane (Fig 2B). Striking differences that might explain the difference in glucose induced insulin release is that islets isolated with lot 2 had much less insulin granules than lot 1 (Fig 2C and 2D; see also data online, www.nanotomy.org/ for batch 1 islets and http://www.nanotomy.org/ for batch 2 islets, login: hIslets password: transplantation for both data sets). A striking difference was the observation that insulin granula in the batch 1 islets were mostly laterally located. They were located near one site of the cell (Fig 2C) and in some cases this was near a capillary. When compared to islets in an intact pancreas (see www.nanotomy.nl) [38] the islet cells were having less cell-cell junctions connecting the cells.

**Fig 3:**
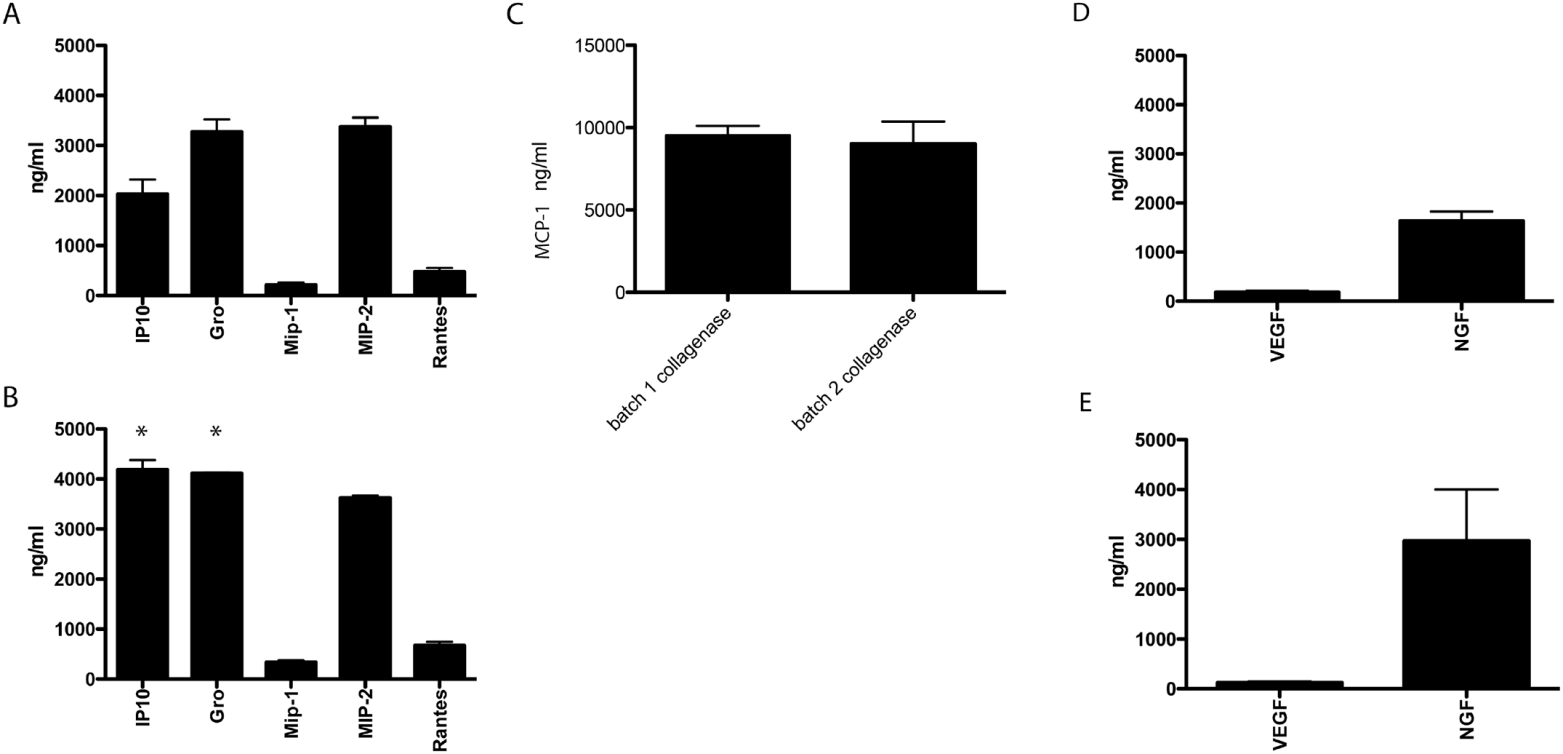
Cytokine, chemokine, and growth factor production by rat-islets is collagenase-type dependent. IP-10, Gro-α, MIP-1, MIP-2, and RANTES production by pancreatic-islets after digestion of the pancreas with collagenase batch 1 (A) or batch 2 (B). MCP-1 production by pancreatic-islets after digestion of the pancreas with collagenase batch 1 or batch 2 (C). VEGF-165 and NGF production by pancreatic-islets after digestion of the pancreas with collagenase batch 1 (D) or batch 2 (E). Values represent mean ± SEM. * indicates statistical significant differences (*p* < 0.05) when compared to batch 1 islets.

Recently it has been shown that collagenase-isolated islet-cells are potent producers of mRNA of several chemotactic chemokines and cytokines [39]. Here we addressed whether this phenomena can be collagenase-lot dependent and also occurs on a protein level. The production of the chemokines IP-10, Gro-α, MIP-1, MIP-2, and RANTES (Fig 3A and 3B) as well as the production of MCP-1 (Fig 3C) and the innervation and vascularization factors NGF and VEGF was compared (Fig 3D and 3E). Both collagenases induced a strong and persistent release of MCP-1 as reported before (Fig 3C) [40], but we also found production of the other chemotactic chemokines (Fig 3). The production of IP-10 and Gro-α are significantly lower with lot 1 when compared to lot 2 collagenase (Fig 3A versus 3B).

### Efficacy of the grafts with different islet quality in curing diabetes

**Fig 4:**
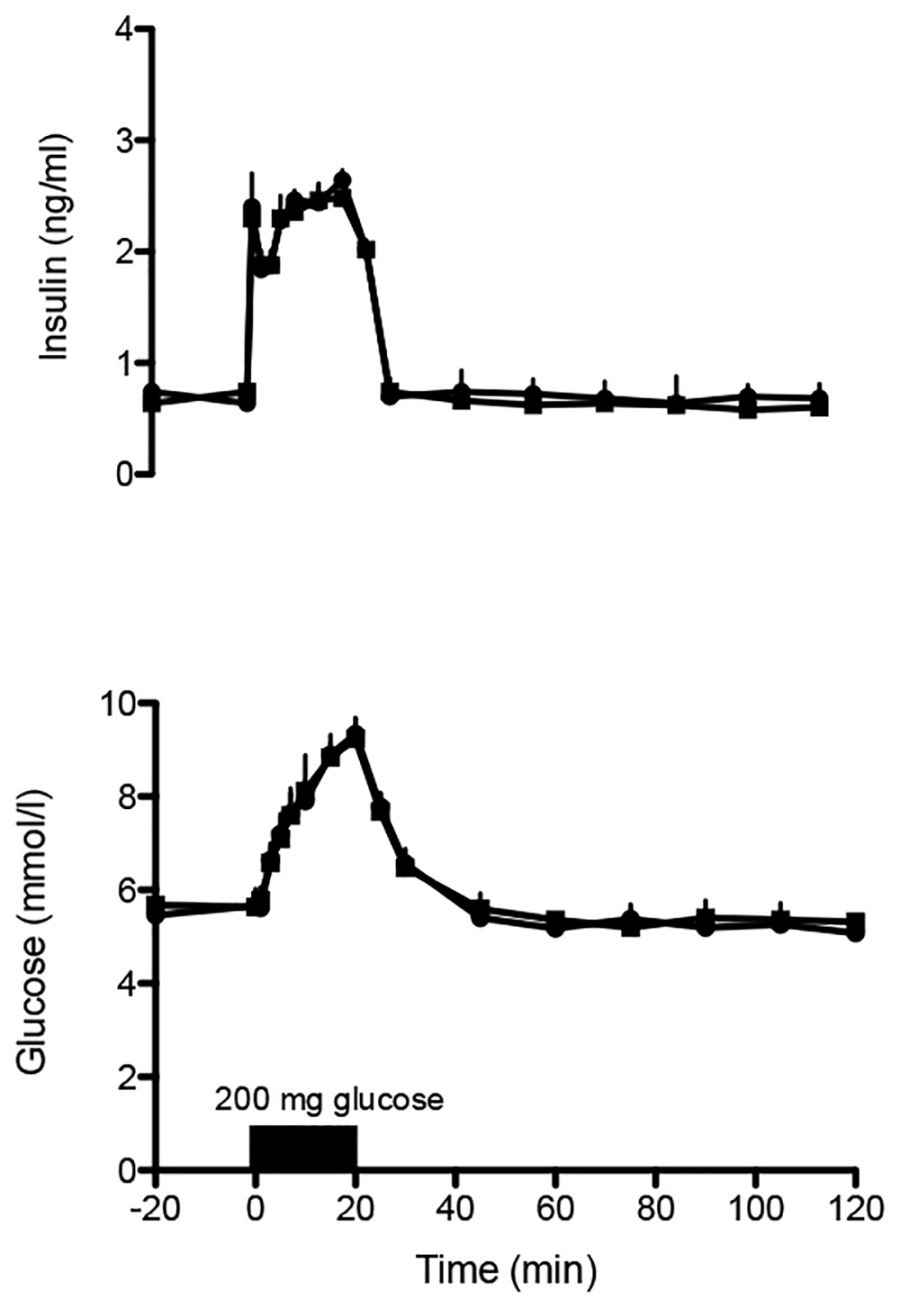
Blood glucose and plasma insulin levels after intravenous glucose infusion (10 mg/min) (n = 5) in AO- recipients of free, nonencapsulated islet grafts of 10 μl of islet tissue, i.e. the equivalent volume of the endocrine pancreas of a rat at four weeks after implantation. Islets were isolated either with batch 1 (circles) or batch 2 (squares). Values represent mean ± SEM.

Next we determined whether the functional, ultrastructural, and immunological differences in the graft let to any effects on graft function or survival [26]. First we determined the efficacy of the two islet grafts in curing diabetes in a free, unencapsulated isograft combination. All recipients (n = 5) received a graft that was composed of 10 μl of islet tissue, i.e. the equivalent volume of the endocrine pancreas of a rat. All recipients returned to normoglycemia within 2 days after transplantation and remained normoglycemic for the entire study period. There were no differences in the time required to restore normoglycemia. To determine whether the differences in islet quality influences the functional capacity of isografts to respond to a glucose-load, we determined the ability of the grafts to respond to an intravenous glucose tolerance test (IVGTT) at four weeks after implantation. There were no differences in efficacy of the grafts in regulating glucose metabolism (Fig 4), illustrating that the graft quality differences were not dramatic and were not detectable after implantation as isografts.

Next we implanted the islet preparations as microencapsulated grafts. All recipients became normoglycemic within 5 days after implantation (n = 7, for batch 1, n = 8 for batch 2). For each group an additional two animals were implanted for examination of the inflammatory environment in the first week after transplantation.

**Fig 5:**
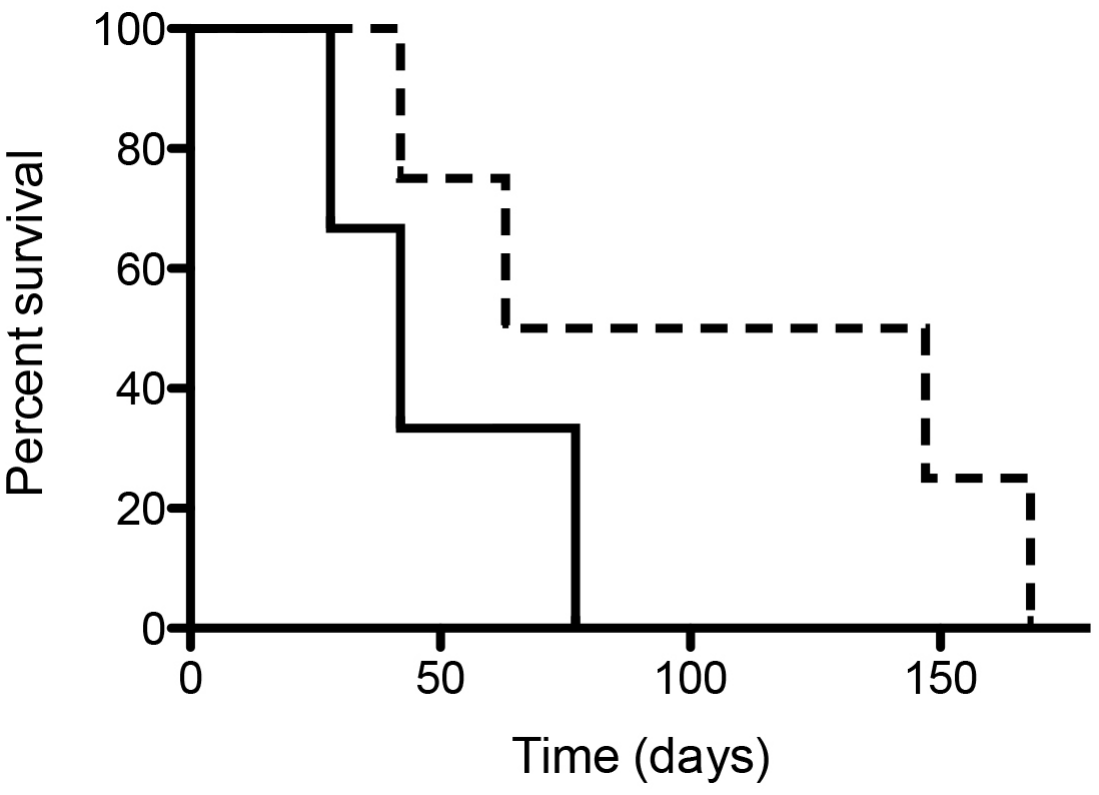
Percentage survival of encapsulated islet allografts of 10 μl microencapsulated islet tissue. Islets were isolated with batch 1 collagenase (broken line) or batch 2 collagenase (see Tables 1 and 2 for details on collagenase batches).

We found no differences in the time required to restore normoglycemia, however, batch 1 survived for 102 ± 15 days while only 61 ± 8.2 days with batch 2 (*p* < 0.02) (Fig 5). In the retrieved grafts less than 10% of the capsules was affected by fibrosis and reported previously [41]

### Differences in the composition of the cellular overgrowth

To assess that the difference in survival and function of the microencapsulated grafts were caused by difference in inflammatory responses against the capsules we performed histology in GMA-embedded sections and immunocytochemistry on frozen sections of the explanted grafts after graft failure and in 2 animals from both groups at 7 days after implantation.

**Fig 6:**
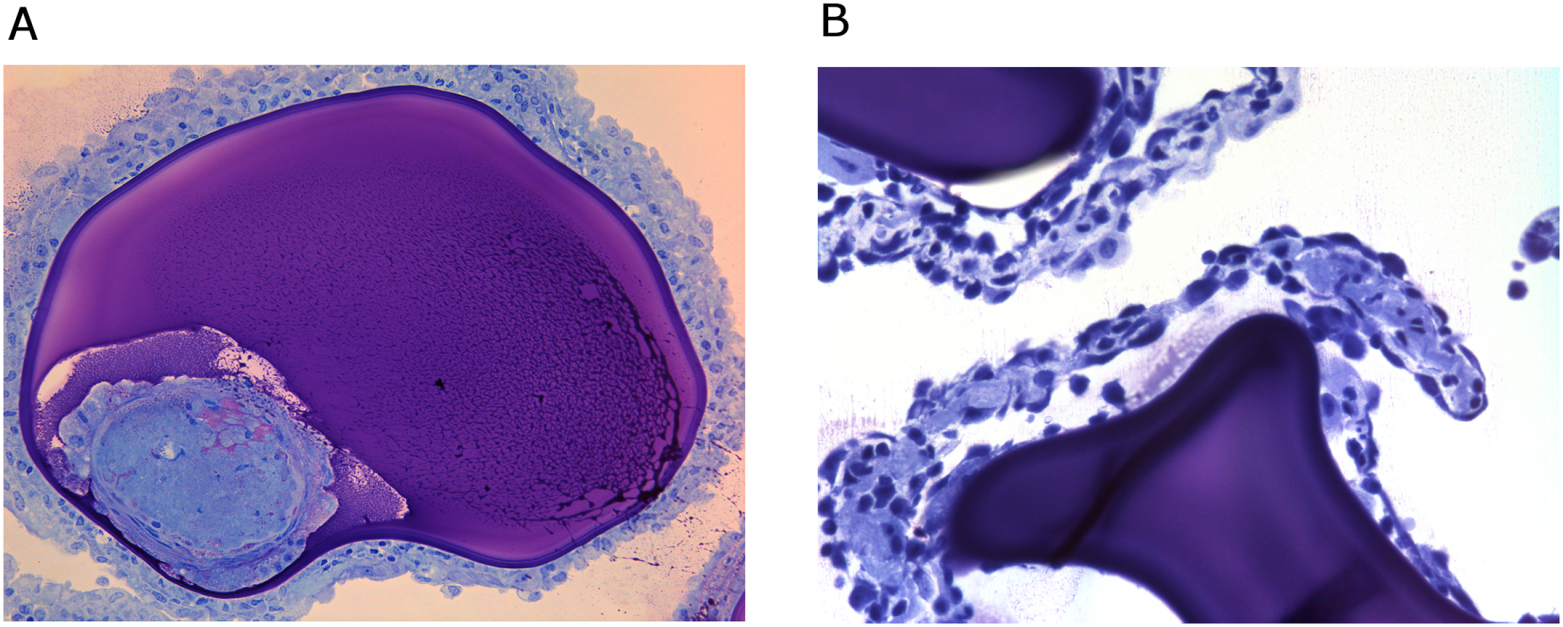
Explanted capsules at 7 days after implantation. A) Clumps of inflammatory cells around capsules containing batch 2 islets. B) An islet-containing capsules from batch 2 islets with a large necrotic zone in the center at 7 days after implantation. Romanovsky-Giemsa staining. Original magnification x 10.

At day 7 most capsules could be flushed out from both groups. Only a few capsules contained cell-adhesion which were all ED1 or ED2 expressing macrophages. However in between the capsules flushed out at day 7 we found more inflammatory cells and clumps of capsules in the batch 2 grafts which were mostly basophils, neutrophils, ED1 and ED2 positive macrophages. Some clumps were also observed in the lavage of the batch 2 grafts but they were rare and much smaller in size suggesting that the batch 1 islets attracted much more inflammatory cells to the grafts. In the clumps of batch 2 grafts we found no surviving islets (Fig 6).

At the time of graft failure we washed out more than 85% of the capsules from the rat peritoneal cavity. There were no statistical differences in retrieval rate between the batches of encapsulated islets. The percentage of overgrown capsules was 8.2 ± 1.2% with batch 1 islets which was similar if not identical to the 7.8 ± 1.4% with batch 2 islets. The cells on this portion of capsules were mostly fibroblasts with scattered a monocytes/macrophages. As in previous studies [15], we found no elements of the specific immune system such as B- or T-lymphocytes.

## Discussion

Many have made efforts to understand the factors leading to success or failure of encapsulated islet grafts [12, 20, 42]. Most of these efforts have been focused on biomaterial-related aspects. Here we show, to the best of our knowledge for the first time, that seemingly minor differences in enzyme activities in collagenase lot numbers has functional, ultrastructural, and immunological consequences for encapsulated islet grafts. Notably, it is not claimed that effects of unfavorable collagenase lots on islet function is new. This is well known [43, 44]. What is novel to our opinion is that even within recommended, accepted boundaries of individual enzyme activities (Table 1) for rat islet isolation [26] with confirmed efficacy in free-rat islet-transplantation studies a profound difference in ultrastructure, immunogenicity and longevity of encapsulated islet-grafts can be observed.

This study was performed with rat-islets as the procedure to isolate rat islets is highly standardized with low variability in yield and viability [26, 28]. This delivers a highly standardized islet source on which we could conveniently test the effect of different enzymes on islet isolation and longevity of free and encapsulated grafts [26]. With other, less standardized islet-sources such as human, dog, baboon, or pig islets, these variations may not be clearly visible, as they will disappear in the background variations caused by other variables. Islet viability and immunogenicity variations might however be important issues in the current debate on the efficacy of encapsulated grafts in large experimental animals such as dogs, pigs, and baboons [45]. Lack of function and low survival rates have been reported in large mammals [45], while others have shown better results [46]. Also, here difference in islet quality may play a role and caution should be taken in interpretation of the results. Several research groups have considerable experience with isolation of islets from rodents and many factors influencing the quality of the preparation have been identified and are standardized to obtain optimal and reproducible islet quality [25]. This is different with large mammals in which the experience is limited and more variation in quality of the grafts is obtained [47, 48]. This leads to more variations in islet quality. We feel that this factor, in addition to differences in capsule properties should be considered as causative for the large, undesired lab-to-lab variations of encapsulated islet grafts in larger mammals. This argumentation is supported by the large species differences in regenerative capacity of islets [49–51]. It is known that rat β-cells do have the capacity to replicate in capsules [52]. Such studies have not been reported for porcine, baboon, or human islets. These islet-related factors should to our opinion been taken into consideration when large mammal studies or studies in humans are performed to investigate the efficacy of encapsulated islets.

Collagenase enzyme preparations with the activities reported in this study have been applied for decades and applied in several free islet-transplantation experiments where they were successfully applied for isolation and transplantation of islets in the liver, spleen, and kidney capsules and in different volumes [26, 53–58]. No adverse effects were found with free rat-islet grafts within these boundaries [26, 53–58] as also confirmed in this study. It was only observed with encapsulated grafts even when the equivalent of the islet-graft volume of the naïve rat-pancreas was applied.

In our design we used the peritoneal cavity for encapsulated grafts and the kidney capsule for free-islet grafts. It can be argued that this is not an adequate comparison but the peritoneal cavity is the only site that can bear the relative large volume of an encapsulated graft [59] but is not an adequate site for free-unencapsulated rat-islets [56]. Therefore, the kidney capsule was chosen for free islet-grafts as it has been shown to allow for comparison of efficacy of islet-grafts in our group [54]. In both encapsulated and free grafts, the equivalent of a rat pancreas was transplanted, 10 μl of islet-tissue, which is known to induce normoglycemia in 100% of the recipients [35, 54] and avoids that differences in metabolic load put on the grafts might be a factor involved in the differences in success rates. The 10 μl is not a marginal dose for islet-grafts in the peritoneal cavity [33] and can to our opinion not be considered as a causal factor for the differences in failure. It might also be argued that isogenic islets had to be used for encapsulated grafts. However, all encapsulated islet grafts do contain protruding islets [60, 61]. These protruding islets may get vascularized, contribute as vascularized graft and interfere with sound interpretation of both the metabolic and longevity data. Therefore we recommend using allografts for these types of studies.

To address ultrastructural effects of collagenase on islets we applied nanotomy that allows for sub-cellular analysis of organelles, granules, and supramacromolecules of the different cells of the islets [38]. It also allows detailed inspection of cell-cell contacts by examining the quality of gap junctions, tight junctions, and desmosomes and is therefore instrumental for judging the effects of islet-isolation procedures on islet quality. As shown here isolation of rat-islets, which belong to the best standardized procedures [26], were all resulting in islets with disturbed intercellular contacts. When compared to islets in the native pancreas (see www.nanotomy.nl) collagenase-isolated islets have much more intercellular spaces and less interconnected areas with tight cell-cell junctions. The ultrastructural effects proved to be collagenase lot dependent as we found that the batch 1 islets which showed better function in the glucose induced insulin release test contained a better polarized organization of the β-cells with lateral insulin-containing granules near the cell membrane. This was not found in batch 2 islets that were not only less organized but also contained much less insulin granules.

Another characteristic of the isolated rat islets was that islets contained cells with swollen vesicles and dark cytoplasm often combined with a damaged plasma membrane indicative for cells in the process of necrosis. This is a rather disturbing observation as cells in the process of necrosis are releasing highly immunogenic intracellular molecules such as danger associated molecular patterns (DAMPs). DAMPs can bind to pattern recognition receptors such as toll-like receptors and induce immune responses [62]. It might even be that by autocrine mechanisms DAMPS are responsible for the high amount of chemotactic cytokines that are being produced by the islets. This DAMP release and/or the elevated cytokine release must be the cause for the different degree of inflammation in the vicinity of the capsules at day 7 after transplantation.

Major differences were found in the production of IP-10 and Gro-α. IP-10 stands for interferon gamma-induced protein 10 and is also called C-X-C motif chemokine 10 (CXCL10). It can be produced by endocrine cells but also by endothelial cells or fibroblasts in the islets [63]. IP-10 is a chemoattractant for monocytes/macrophages, T cells, NK cells, and dendritic cells [64, 65]. Gro-α is the same cytokine as CXCL1 and was previously called neutrophil-activating protein 3. It is produced by epithelial cells and has neutrophil chemoattractant activity [66, 67]. The difference in production of these chemokines by the islets is very likely the cause of the different degree of inflammation that was observed in the peritoneal cavity in the vicinity of the capsules at day 7 after transplantation. In this first phase, directly after implantation, significant amounts of islets can be lost due to inflammatory responses [37, 68]. It was not known however that this was influenced by islet-derived factors as well.

A principle difference that should explain the differences in findings between encapsulated and free islet-grafts is that free, nonencapsulated islets are vascularized within days after implantation and will recover from the isolation stress within the first weeks after implantation [69, 70]. The damage that is caused by the enzymatic digestion is related to the breakdown of the extracellular matrix within the islets. After implantation microencapsulated islets are not revascularized but instead exposed to an inflammatory response associated with implantation surgery [68, 71] and as shown here reinforced by islet-derived factors such as chemokines. This response can take up to two weeks [68] during which substantial damage can be done to the islets [37]. Islets in non-optimal condition will have less chance to accommodate to these *in vivo* circumstances and will not survive this period. For these reasons it is of essential importance to apply the highest islet-quality possible for encapsulation procedures. Our recommendation would be not to use collagenase preparations with enzyme activities above the activities mentioned for batch 1 in Table 1. Especially neutral protease and clostripain activity should be kept as low as possible in collagenases applied for islets to be encapsulated.

Documentation of the activities of the applied enzyme solutions may lead to side-by-side comparison and a better understanding of the factors contributing to the reported lab-to-lab variations in success of encapsulated islet grafts [20, 42, 72] which is considered to be a major obstacle in application of encapsulated islets-grafts [20, 42, 72]. To the best of our knowledge this has not happened in any report involving encapsulated islets up to now. Now clinical studies are being started it is essential to document all these parameters in order to understand what has contributed to success or failure of the trials.

In conclusion, we show that besides the physicochemical characteristics of the immunoisolating devices also the ultrastructural and immunological properties of the enveloped tissue impact long-term functional survival. The quality and characteristics of islets in immunoisolated grafts are rarely documented but is essential for understanding the factors determining success or failure of immunoisolated islet grafts. An additional important conclusion is to our opinion that our study highlight that relative small variations in producing encapsulated-islets in the same facility can lead to distinct efficacies *in vivo*.
